# Risk Factors for Preterm Birth in an International Prospective Cohort of Nulliparous Women

**DOI:** 10.1371/journal.pone.0039154

**Published:** 2012-07-16

**Authors:** Gustaaf Albert Dekker, Shalem Y. Lee, Robyn A. North, Lesley M. McCowan, Nigel A. B. Simpson, Claire T. Roberts

**Affiliations:** 1 Lyell McEwin Hospital, Adelaide, South Australia, Australia; 2 Robinson Institute, University of Adelaide, Adelaide, South Australia, Australia; 3 Division of Women’s Health, Women’s Health Academic Centre, King’s College London and King’s Health Partners, London, United Kingdom; 4 Department of Obstetrics and Gynaecology, University of Auckland, Auckland, New Zealand; 5 Academic Department of Obstetrics and Gynaecology, University of Leeds, Leeds, United Kingdom; Tehran University of Medical Sciences, (Islamic Republic of Iran)

## Abstract

**Objectives:**

To identify risk factors for spontaneous preterm birth (birth <37 weeks gestation) with intact membranes (SPTB-IM) and SPTB after prelabour rupture of the membranes (SPTB-PPROM) for nulliparous pregnant women.

**Design:**

Prospective international multicentre cohort.

**Participants:**

3234 healthy nulliparous women with a singleton pregnancy, follow up was complete in 3184 of participants (98.5%).

**Results:**

Of the 3184 women, 156 (4.9%) had their pregnancy complicated by SPTB; 96 (3.0%) and 60 (1.9%) in the SPTB-IM and SPTB-PPROM categories, respectively. Independent risk factors for SPTB-IM were shorter cervical length, abnormal uterine Doppler flow, use of marijuana pre-pregnancy, lack of overall feeling of well being, being of Caucasian ethnicity, having a mother with diabetes and/or a history of preeclampsia, and a family history of low birth weight babies. Independent risk factors for SPTB-PPROM were shorter cervical length, short stature, participant’s not being the first born in the family, longer time to conceive, not waking up at night, hormonal fertility treatment (excluding clomiphene), mild hypertension, family history of recurrent gestational diabetes, and maternal family history of any miscarriage (risk reduction). Low BMI (<20) nearly doubled the risk for SPTB-PPROM (odds ratio 2.64; 95% CI 1.07–6.51). The area under the receiver operating characteristics curve (AUC), after internal validation, was 0.69 for SPTB-IM and 0.79 for SPTB-PPROM.

**Conclusion:**

The ability to predict PTB in healthy nulliparous women using clinical characteristics is modest. The dissimilarity of risk factors for SPTB-IM compared with SPTB-PPROM indicates different pathophysiological pathways underlie these distinct phenotypes.

**Trial Registration:**

ACTR.org.au ACTRN12607000551493

## Introduction

In the developed world, spontaneous preterm birth (SPTB) is without doubt a major problem in modern obstetrics; its prevalence is still rising in many industrialised countries. According to the USA National Vital Statistics Reports, 11–12% of the 4 million neonates born each year are delivered before 37 weeks and 3.6% are delivered before 34 weeks [1]–[3]. Early PTB (before 34 weeks) is particularly associated with high rates of mortality and morbidity, including intraventricular hemorrhage, necrotizing enterocolitis, respiratory distress syndrome and neurological deficit [2]. PTB has long-term medical and social sequelae; the risk of medical and social disabilities in adulthood increases with decreasing gestational age at birth [4], [5].

To identify women at risk of SPTB, clinicians use prior preterm birth, multiple pregnancy and prior cervical surgery as major risk factors. Useful clinical risk factors in predicting SPTB in nulliparous women with a singleton pregnancy are scant, except for a history of prior cervical surgery. In low risk women, maternal history alone misses more than half of the women at risk for SPTB [6]. The use of vaginal posterior fornix testing for fetal fibronectin only yields meaningful positive tests after 22 weeks gestation and may be only a few weeks prior to the actual preterm birth. Measuring cervical length is the only screening test for SPTB that has been shown to have potential for effective intervention. Fonseca et al. [7] demonstrated, in a cohort of seemingly low risk women with cervical length ≤1.5 cm at 20 weeks gestation (n = 413), that vaginal progesterone reduced the risk of SPTB by 45%. While most countries have not introduced routine screening for cervical shortening in asymptomatic patients, a recent cost-effectiveness analysis concluded that on the basis of the published efficacy of vaginal progesterone treatment, cervical length measurement should become part of routine antenatal care [8].

It is important to note that ‘preterm birth’ is in itself not a diagnosis. The term describes the clinically easily identifiable end-result of various different major pathophysiological pathways. Preterm labour leading to SPTB may present with intact membranes (SPTB-IM) or following spontaneous rupture of membranes (SPTB-PPROM); the pathways leading to these different clinical phenotypes are likely to be different [9].

The SCOPE (Screening for Pregnancy Endpoints) study is a prospective, multi-centre cohort study of ‘healthy’ nulliparous women, with the primary aim of developing screening tests to predict preeclampsia, small for gestational age (SGA) infants and SPTB. The study design incorporated prospective collection of information on all known clinical risk factors for preterm birth.

The objectives for this part of SCOPE were to identify risk factors for SPTB-IM and SPTB-PPROM and to develop multivariable predictive models based on clinical risk factors present in early pregnancy (15±1 weeks), together with cervical length measurements and routine sonographic findings obtained during the ‘morphology scan’ at 20±1 weeks’ gestation.

**Figure 1:**
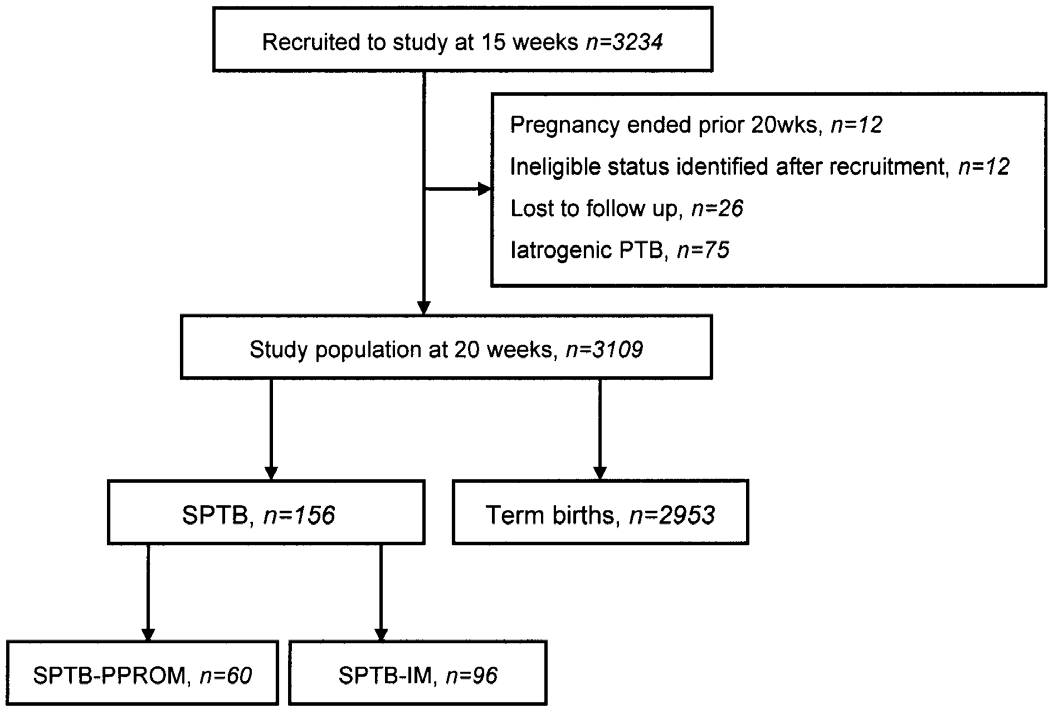
Participants recruited and study population.

**Table 1:** Clinical characteristics.

	Term births	SPTB-IM	P	SPTB-PPROM	P
**Maternal Characteristics**	**2953**	**96**		**60**	
Age	28.0 (5.8)	27.6 (6.5)	0.50	28.0 (5.8)	0.90
BMI	25.6 (5.3)	26.1 (5.5)	0.35	25.2 (6.0)	0.58
Height (cm)	165.2 (6.6)	164.5 (6.9)	0.26	163.3 (6.7)	***0.023***
Head circumference (cm)	56.0 (1.7)	55.9 (1.4)	0.47	55.5 (1.6)	***0.019***
Systolic BP (mmHg)	108 (11)	108 (10)	0.95	107 (11)	0.55
Diastolic BP (mmHg)	65 (8)	66 (8)	0.31	65 (8)	0.86
Caucasian	2558 (86.6)	90 (93.6)	***0.048***	52 (86.7)	0.99
First born	1708 (58.1)	42 (44.2)	0.66	15 (25.0)	***0.01***
**Social Characteristics**					
SEI	40.674 (16.5)	39.5 (17.3)	0.51	40.3 (15.1)	0.87
Full-time employment	1972 (66.8)	58 (60.4)	0.19	44 (73.3)	0.29
**Diet Characteristics**					
Smoking (15 weeks)	313 (10.6)	22 (22.9)	***0.000***	9 (15.0)	0.28
Marijuana (pre-preg)	191 (6.5)	17 (17.7)	***0.000***	5 (8.3)	0.57
Marijuana (1st trimester)	31 (1.0)	8 (8.3)	***0.000***	2 (3.3)	0.11
**Psychological Characteristics**					
Anxiety Index >90%	211 (7.2)	12 (12.6)	***0.049***	6 (10.0)	0.41
Not feeling better than ever	2275 (77.5)	83 (86.5)	***0.04***	48 (80.0)	0.64
**Obstetric Characteristics**					
Gravidity	1.3 (0.6)	1.6 (0.8)	***0.000***	1.4 (0.6)	0.54
Months to conceive	5.9 (11.6)	7.4 (11.9)	0.23	11.9 (22.1)	***0.000***
< = 3 months to conceive	1871 (63.6)	51 (53.1)	***0.038***	31 (51.7)	0.06
Donor sperm	141 (4.8)	5 (5.2)	0.84	8 (13.3)	***0.004***
Hormonal treatment	90 (3.0)	2 (2.1)	0.59	7 (11.7)	***0.001***
Mild Hypertension (not on treatment)	29 (1.0)	2 (2.1)	0.30	3 (5.0)	***0.007***
LLETZ	107 (3.6)	7 (7.3)	0.07	7 (11.7)	***0.002***
>1 Vaginal bleeding	145 (4.9)	9 (9.4)	0.05	4 (6.7)	0.54
APH	162 (5.5)	23 (24.0)	***0.000***	5 (8.6)	0.31
Waking at night					
Once	918 (31.2)	27 (28.1)	0.10	13 (21.7)	***0.014***
≥2 times	1837 (62.5)	59 (61.5)	0.13	39 (65.0)	0.07
Cervical length (mm)	41.0 (7.4)	38.7 (7.9)	***0.006***	38.9 (6.9)	***0.047***
Average UTRI >90%	240 (7.5)	17 (18.1)	***0.002***	7 (12.7)	0.27
Average UTRI	0.56 (0.09)	0.59 (0.12)	***0.002***	0.57 (0.09)	0.29
**Family History**					
Gestational diabetes	106 (3.6)	8 (8.3)	***0.020***	5 (8.3)	0.062
Recurrent GDM	19 (0.6)	2 (2.1)	0.11	2 (3.3)	***0.027***
Preeclampsia	284 (9.6)	20 (20.8)	***0.000***	5 (8.3)	0.74
Mother had preeclampsia	233 (7.9)	16 (16.7)	***0.003***	4 (6.7)	0.73
Gestational Hypertension	6 (0.2)	0 (0.0)	0.98	1 (1.7)	***0.051***
Miscarriage (mother)	888 (30.1)	28 (29.2)	0.85	10 (16.7)	***0.028***
Diabetes Type 2 (mother)	137 (4.6)	9 (9.4)	***0.037***	2 (3.3)	0.63
Low birthweight baby*	27 (0.9)	5 (5.2)	***0.000***	1 (1.7)	0.55
**Birth Outcomes**					
Gestational age 40 (1)		34 (4)	0.97	33 (5)	0.97
Birthweight (g)	3481 (472)	2378 (736)	***0.000***	2379 (761)	***0.000***
Customized centile	49 (29)	49 (31)	0.85	51 (32)	0.50
SGA	285 (10)	11 (11.5)	0.56	6 (10)	0.93

Characteristics as mean (SD) or n (%); head circumference and height in centimetres; ^x^ mother/sister with low birth weight baby; APH  =  antepartum haemorrhage; BP  =  blood pressure; UTRI  =  uterine artery resistance index.

## Methods

The STROBE checklist for this trial is available as supporting information; see Checklist S1.

Nulliparous women with singleton pregnancies were recruited to the SCOPE study between November 2004 and August 2008 in Auckland, New Zealand, and Adelaide, Australia. Ethical approval was obtained from local ethics committees [New Zealand AKX/02/00/364, Australia REC 1712/5/2008] [10] and all women provided written informed consent.

Women attending hospital antenatal clinics, obstetricians, general practitioners or community midwives prior to 15 weeks’ gestation were invited to participate in the SCOPE study. Women were excluded if (1) they were judged to be at a particularly high risk of pre-eclampsia, SGA or SPTB due to underlying medical conditions (chronic hypertension requiring antihypertensive medication, diabetes, renal disease, systemic lupus erythematosus, anti-phospholipid syndrome, sickle cell disease, human immunodeficiency virus), previous cervical knife cone biopsy, ≥3 terminations or ≥3 miscarriages, current ruptured membranes; 2) their pregnancy was complicated by a known major fetal anomaly or abnormal karyotype or 3) they had received interventions that might have modified pregnancy outcome (e.g., aspirin, cervical suture) [10]. Participants were interviewed and examined by a research midwife at 15±1 and 20±1 weeks of gestation and underwent an ultrasound scan at 20±1 weeks. At the time of interview, data were entered into an internet accessed, central database with a complete audit trail (MedSciNet^AB^).

At time of recruitment the following data were collected [10]: demographic information including age, ethnicity, immigration details, education, work, socioeconomic index, income level, living situation; the woman’s birthweight and gestation at delivery, and whether it was a singleton or multiple pregnancy; previous miscarriages, terminations or ectopic pregnancies and whether these pregnancies were with the same partner as the current pregnancy or not; history of infertility, use of assisted reproductive technologies, duration of sexual relationship and exposure to partner’s sperm; gynaecological (number of cervical dilatations, abnormal PAP smears, and treatment for cervical intraepithelial neoplasia, polycystic ovarian syndrome) and medical history including hypertension while taking combined oral contraception, asthma, urinary tract infection, inflammatory bowel disease, thyroid disease and thrombo-embolism; family history (mother, sisters) of obstetric complications (miscarriage, preeclampsia, eclampsia, gestational hypertension, spontaneous preterm birth, any preterm birth, gestational diabetes, stillbirth and neonatal death) and family history (mother, father, sibling) of medical conditions (hypertension, coronary artery heart disease, cerebrovascular accident, type 1 and 2 diabetes and venous thrombo-embolism).

Information was collected on early pregnancy vaginal bleeding (gestation, severity and duration of bleeding and number of bleeding episodes), hyperemesis and infections during pregnancy. Vegetarian status was recorded and other dietary information pre-conception and during pregnancy was obtained using food frequency questions for fruit, green leafy vegetables, oily and other fish and fast foods. Use of folate and multivitamins, cigarettes, alcohol (including binge drinking) and recreational drugs (including marijuana, amphetamine, cocaine, heroin, ecstasy, lysergic acid diethylamide) was recorded for preconception, 1^st^ trimester and at 15 weeks. A lifestyle questionnaire was completed on work, exercise and sedentary activities, snoring, domestic violence and social support. Psychological scales were completed measuring perceived stress, depression, anxiety, and behavioural responses to pregnancy (adapted from the Behavioural Responses to Illness Questionnaire [11]). Two consecutive manual blood pressure measurements were recorded. Other maternal measurements included maternal height, weight and waist, hip, arm and head circumference.

Ultrasound examination at 20±1 weeks’ gestation included measurements of the fetus (biparietal diameter, head circumference, abdominal circumference and femur length), Doppler studies of the umbilical and uterine arteries, and transvaginal cervical length measurements [12]. Notching of each uterine artery was recorded. An abnormal uterine artery.

Doppler result was defined as a mean resistance index >90th centile (>0.695) [12].

The technique used to measure the cervical length was that modified from Berghella et al. [13].

As described by Gomez et al [14] no fundal or suprapubic pressure was applied during the examinations. All fetal measurements were adjusted for gestational age by calculating the multiple of the median for each gestational week.

Participants were followed prospectively, with pregnancy outcome data and baby measurements collected by research midwives. Data monitoring included 1) individually checking all data for each participant, including any transcription errors of the lifestyle questionnaire, and 2) detection and correction of illogical or inconsistent data and outliers using customised software.

Primary outcome: The primary outcome was SPTB (birth <37 weeks’ gestation) as per the two main phenotypes, i.e. SPTB-IM and SPTB-PPROM. SPTB-PPROM was defined as SPTB where the women presented with confirmed rupture of the membranes in the absence of labour and the time between the rupture of the membranes to delivery was at least 6 hours greater than the combined time for established labour (i.e. duration of first stage + duration of second stage [10]).

**Table 2:** Clinical risk factors at 15 weeks, and ultrasound scan variables at 20 weeks in logistic regression model for SPTB-IM.

SPTB-IM			
	OR	Lower95%	Upper95%
BMI <20	1.46	0.62	3.42
BMI 25–30	1.63	0.96	2.79
BMI >30	1.21	0.63	2.32
Caucasian ethnicity	2.73	0.98	7.60
Marijuana pre-pregnancy	2.34	1.22	4.52
Not feeling better than ever	1.78	0.90	3.51
Having a history of >1 vaginal bleed	2.33	1.08	5.04
Mother with diabetes type 1 or 2	2.19	0.99	4.86
Mother with a history of preeclampsia	2.34	1.30	4.21
Strong family history of low birthweight babies	5.64	1.79	17.80
Abnormal uterine artery Doppler20 wks	2.18	1.20	3.94
Shortest transvaginal cervical lengthin mm	1.05	1.01	1.08

Reference BMI 20–<25.

**Table 3:** Clinical risk factors at 15 weeks, and ultrasound scan variables at 20 weeks in logistic regression model for SPTB-PPROM.

SPTB-PPROM			
	OR	Lower 95%	Upper 95%
BMI <20	2.64	1.07	6.51
BMI 25–30	1.20	0.57	2.51
BMI >30	0.94	0.39	2.26
Height (per cm)	0.93	0.89	0.97
Participant position in family	1.91	0.97	3.76
Waking once a night	0.32	0.12	0.89
Waking more than once a night	0.45	0.19	1.05
Months to conceive (per month)	1.02	1.00	1.03
Other hormonal fertility treatment^1^	3.67	1.24	10.83
Mild hypertension not requiring treatment	9.65	2.51	37.14
Family history of recurrent GDM^2^	8.01	1.51	42.45
Maternal family history of any miscarriage	0.43	0.19	0.94
Shortest transvaginal cervical length per mm	1.05	1.01	1.09

1 =  hormonal fertility treatment other than clomiphene; GDM  =  gestational diabetes mellitus; participant’s position in family  =  index mother not being the first-born); Reference BMI 20–<25.

### Statistical Methods

Women who had SPTB-IM or SPTB-PPROM were separately analyzed and compared with all women who had term births. Variables with more than 10% missing data were excluded from analyses, with the exception of the dental health variables included in the univariate analysis only (available in 38% of participants as added later to the database) and cervical length in the multivariable analysis. Of the variables selected for modelling, data were complete in >99% of participants for each variable other than cervical length (18.6% missing data), uterine artery Doppler (5% missing) and participant born preterm before 34 weeks’ gestation (4% missing). Missing data was handled in the multivariable analysis by omitting participants with any missing data. R version 2.12.1 was used to perform the analyses. Univariate data analyses including Student’s *t* test and Chi-square tests were used to compare and test the association of predictors with SPTB-IM and SPTB-PPROM.

A total of 933 variables were tested for association with SPTB-PPROM and SPTB-IM separately using univariate analysis. Variables were then excluded due to P value >0.1 on univariate comparison (797 variables for SPTB-PPROM, 691 variables for SPTB-IM), variables with >10% missing data (5 variables for SPTB-PPROM, 11 variables for SPTB-IM), and variables assessed after 15 weeks of gestation with the exception of uterine artery resistance index and cervical length both measured at 20 weeks of gestation (65 variables for SPTB-PPROM, 87 variables for SPTB-IM). Of the remaining variables, a list of 49 variables for SPTB-PPROM and 30 variables for SPTB-IM were selected based on known predictors and variables of interest. The initial variable lists used to train the multivariate models are available as supporting information (File S1). Two multivariable logistic regression models were then trained for SPTB-PPROM and SPTB-IM based on corresponding selected predictors. A backward stepwise method was used to develop an optimal model. Akaike Information Criteria (AIC) were obtained for each model as a goodness of fit measure and the optimal model was determined based on minimum AIC [15]. The sensitivity and specificity were calculated as measures of goodness of classification. The receiver operating characteristic (ROC) curve and the Area Under Curve (AUC) [16] were also obtained to assess predictive utility. Ten-fold cross validations were performed on all models using 90% of the data randomly chosen for training purposes, and validating on the remaining 10%.

**Figure 2:**
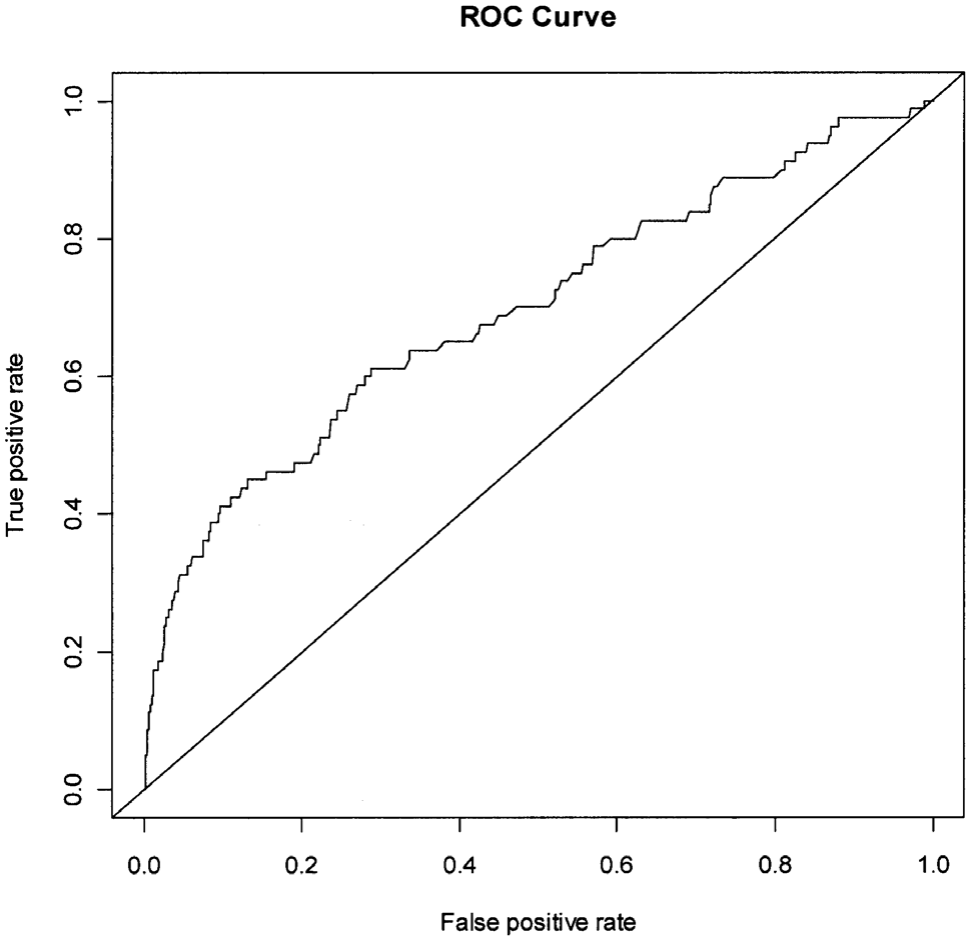
Receiver-operating characteristic curve for SPTB-IM.

**Figure 3:**
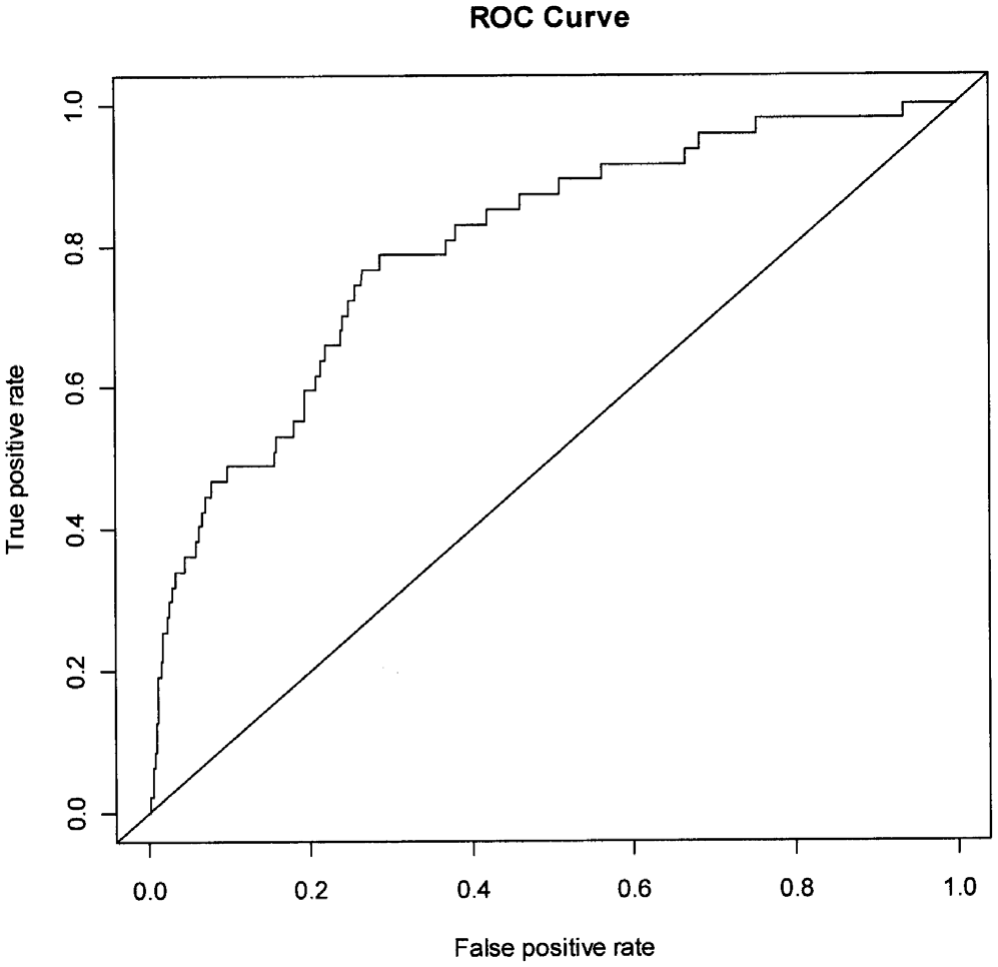
Receiver-operating characteristic curve for SPTB-PPROM.

## Results

3234 nulliparous pregnant women with singleton pregnancies were recruited to the SCOPE study between November 2004 and August 2008 in Auckland, New Zealand and Adelaide, Australia. Follow up was complete in 3184 (98.5%) of participants (Figure 1). Of the total of 156 SPTB, 96 (61.5%) were in the SPTB-IM and 60 (38.5%) in SPTB-PPROM categories. Women with iatrogenic PTB were excluded from the study population.

After omitting participants with any missing data, a total of 2499 (80.4%) patients for SPTB-IM and 2455 (79%) patients for SPTB-PPROM were included in the logistic regression analyses.

The characteristics in this cohort of nulliparous pregnant women with term birth, and the 2 main subtypes of SPTB are shown in Table 1.

In the 1987 participants in whom data on dental health were collected, there was no difference in a history of easily bleeding gums, swollen gums or sore teeth prior to or during the first trimester of pregnancy between the term birth group and either SPTB-IM and SPTB-PPROM.

Clinical risk factors recorded at 15 weeks’ gestation and the ultrasound scan results from the 20 weeks’ gestation, with significant independent associations for SPTB-IM and SPTB-PPROM, and/or contributing to the model are shown in Table 2 and 3, respectively.

In the logistic regression model for SPTB-IM, a shorter cervical length as a continuum was associated with an increased risk of 1.04 per mm decrease in cervical length. Regular marijuana use up to conception was a significant and strong risk factor. Similar risks were found to be associated with the presence of an abnormal uterine Doppler flow velocity waveform pattern at 20 weeks’ gestation and maternal family history of any type of diabetes and/or preeclampsia. A strong family history of low birth weight babies (mother and/or sister with a low birth weight baby) was the strongest risk factor with odds exceeding 5. With regard to a history of vaginal bleeding, only the presence of more than one episode of vaginal bleeding was an independent risk marker. ‘Not feeling better than ever’ contributed to the model for SPTB-IM, though the odds ratio crossed unity (odds ratio 1.78; 95% CI 0.90–3.51).

Whilst Caucasian ethnicity and a low or elevated BMI were included as independent risk factors in the model, the confidence intervals for each adjusted OR crossed unity.

Except cervical length, the independent variables in the SPTB-PPROM model (table 3) were strikingly different to those in the SPTB-IM model. Having a low BMI had an odds ratio of 2.64. For every cm maternal height increase there was a 7% reduced risk for SPTB-PPROM. Length of sexual cohabitation in months, as a continuum, increased the risk by one percent per additional month. Having a history of hormonal fertility treatment (excluding clomiphene), and having mild hypertension (chronic hypertension requiring treatment was an exclusion criterion for the SCOPE study) were both independent risk factors. Having a family history of recurrent gestational diabetes was strongly associated with SPTB-PPROM, albeit with large confidence intervals. Participant’s position in family (index mother not being the first-born) was a significant independent risk factor.

The predictive capability for SPTB-IM is shown in figure 2; AUC 0.69, with a sensitivity of 0.39 based on 90% specificity. Figure 3 shows the ROC curve for SPTB-PPROM; AUC 0.79, with a sensitivity of 0.49 based on 90% specificity.

## Discussion

Analysis of data from this large prospective cohort of low-risk nulliparous pregnant women have demonstrated that clinical risk factors, including cervical length and uterine artery Doppler ultrasound measurements at 20 weeks, have only a modest predictive capacity for the two major phenotypes of SPTB. In this particular analysis we selected a case-control approach instead of a case – non case approach because of potential overlap in pathophysiology not only between the 2 major phenotypes but also between iatrogenic preterm birth and SPTB. Most likely, a strict case-non case approach would have further dropped the performance of the models. While it is clear that these risk markers by themselves cannot be translated into a useful clinical tool for daily practice, the data provide further insight into these conditions.

The minimal overlap between risk factors for SPTB-PPROM and SPTB-IM reinforces the increasingly accepted view that SPTB is a heterogeneous entity with different pathological pathways leading to SPTB with or without intact membranes [9] and also differences between patients with SPTB at different gestational ages [17]–[19]. This heterogeneity is illustrated by the observation that antepartum haemorrhage (APH) is significantly more common in the SPTB-IM group (24%) than the SPTB-PPROM group (8.6%) or term births (5.5%). APH was not entered in the multivariate analysis since it occurs by definition after 20 weeks’ gestation.

Regarding variables related to placentation, we found a lengthier sexual relationship (as a continuum) known to exert a protective effect for preeclampsia and intra-uterine growth restriction [20], to be associated with a small but significant increased risk for SPTB-PPROM. It should be noted that in univariate analysis (table 1), conceiving within 3 months (table 1) was also less common in both SPTB phenotypes compared with term birth (SPTB-IM p = 0.038; SPTB-PPROM p = 0.06). In contrast, donor insemination was significantly (p = 0.005) more common in the SPTB-PPROM group (8 out of 60; 13.3%) versus term birth (4.8%). While, the presence of abnormal uterine Doppler flow patterns at the time of the morphology scan nearly doubled the risk for SPTB-IM this was not an independent risk factor for SPTB-PPROM. Also recurrent vaginal bleeding in early pregnancy, a previously described risk factor [21], while doubling the risk for SPTB-IM was not a risk factor for SPTB-PROM.

Decreased cervical length (per mm decrease) was the only variable with a comparable effect in both SPTB phenotypes; 4 and 5% increased risk for SPTB-IM and SPTB-PPROM, respectively. This would mean that for example the risk for SPTB for two comparable nulliparous pregnant women with cervical length of 41 mm versus 28 mm at 20 weeks gestation would be at least 60% higher in the woman with the shorter cervix. Using a cost-effectiveness analysis, Werner et al [8] predicted if there were universal cervical length screening, there would be a net health improvement of 735 quality adjusted life years and net savings to the healthcare system (USA data) of $19 million for every 100 000 women screened. This cost-effectiveness analysis was primarily based on the Fonseca et al [7] study, but the results were analysed and confirmed by including the recent result of the Hassan et al multicentre study [22]. Unfortunately, these 2 large multicentre vaginal progesterone studies do not specifically address the SPTB phenotype.

Most of the independent risk factors for SPTB-IM could, at least in theory, fit in one of the seven major molecular pathways previously described by Romero et al [23]. ‘Not feeling as well’ could be a marker of stress or lack of support, and as such fits in one of the pathways to preterm birth [23]. In contrast to several epidemiological studies on stress and employment [24], [25], the other variables capturing data on employment, income, anxiety and depression were not independent risk factors.

We have shown that marijuana is a strong ‘environmental risk factor or SPTB-IM in this population. We are unable to determine whether this association is due to a toxic effect of marijuana or is a marker of a suite of lifestyle factors that contribute to the risk. Pre-pregnancy marijuana use may be a more reliable marker since one can anticipate that women would be more likely to disclose it than persistent marijuana use during pregnancy. In contrast to the results of this large prospective cohort study, large American population studies [26]–[28], did not find an association between maternal marijuana use and preterm birth.

In this cohort of 3234 low risk nulliparous women, with 156 cases of SPTB, we do find the highest rate of smokers amongst the SPTB-IM group (22.9% versus 10.6% in term births; p 0.00), with an intermediate rate in the SPTB-PPROM group (15%; p 0.291). However, smoking was not an independent risk factor for either phenotype. Because of our very rich data it is possible that the effect of smoking is now explained by other variables in the models such as abnormal uterine artery Doppler [29]. Maternal tobacco smoking has typically been described as a risk factor for SPTB in many studies; however the mechanism for this effect remains unclear [30]. In a retrospective cohort study covering all preterm births in the major tertiary referral centre in Western Australia during the period 2004–2008, Henderson et al [31] found a significant association of smoking in only one SPTB subtype: SPTB-PPROM between 27 and 33 weeks’ gestation, and suggested that these data indicate that tobacco smoking may have a specific effect on the fetal membranes while not influencing spontaneous labour. Furthermore, an analysis based on a large Swedish population cohort [30] demonstrated that smoking (≥10 cigarettes per day; odds ratio 1.7) was primarily associated with increased risks of very preterm birth and there were small numbers of very preterm births in this cohort.

Ethnic differences in the prevalence of various adverse pregnancy outcomes, including SPTB, have been previously described [32], [33]. Although specific high risk genetic polymorphisms may partially explain those ethnic differences, most studies appear to point to socio-economic deprivation, smoking, obesity, poverty-induced stress and the associated poor nutrition as the key mediators. It should be noted that the non-Caucasian pregnant women in this SCOPE cohort consisted primarily of women of Asian descent and to a lesser degree also Maori and Pacific Island women. The low total number of non-Caucasian ethnicities did not permit further sub-analysis. Surprisingly (on univariate comparison) Caucasian ethnicity was significantly more common in the SPTB-IM group. Being of Caucasian ethnicity, as an independent variable in the regression model, more than doubled the risk for SPTB-IM, although the 95% CI just crossed 1. Although this was not captured by our socio-economic variables, these findings might be explained by the fact that women in the Australian part of the SCOPE study come from one of the most underprivileged urban areas in Australia with a primarily Caucasian population [34], [35]. Our data demonstrate that taking a full family history can provide potentially important indicators for risk for SPTB, as a strong family history of low birth weight babies was the strongest risk factor with odds exceeding 5 (albeit present in just over 1% of the whole cohort) for SPTB-IM, while a positive family history in the mother for preeclampsia and any type of diabetes more than doubled the risk.

In addition to decreased cervical length, BMI was the only variable present in both models. Conventional wisdom indicates that women with low BMI are at increased risk for SPTB, while the association between maternal overweight or obesity and SPTB remains controversial. Heterogeneity in the definitions of pregnancy outcomes (spontaneous vs. medically indicated PTB) and the inclusion of different gestational ages in delivery categories in various studies are probably only a partial explanation for these controversies [36]. In this prospective cohort low BMI, doubled the risk for SPTB-PPROM with the odds ratio just crossing 1 (odds ratio 2.1; 95% CI 0.93–4.54). It is not surprising that the contemporary literature regarding BMI and the risk for preterm birth, and indeed any adverse pregnancy outcome, is often conflicting. In the past low BMI was associated with undernutrition. However, more recently obesity has become a marker of socio-economic deprivation with overconsumption of calorie-dense but nutrient-poor food [34], [35].

In contrast to the independent risk factors associated with SPTB-IM, those associated with SPTB-PPROM are largely difficult to explain, and considering the number of variables in the final analysis for SPTB-PROM (49 variables) could well represent false discoveries for some of these findings.

To our knowledge, these data are the first to suggest that greater maternal height only provides protection from SPTB-PPROM but not SPTB-IM. Chan et al [37] previously reported that Asian women of shorter stature were at a higher risk of preterm birth. Transgenerational reproductive adaptation, i.e. earlier birth to allow safe passage through a smaller pelvis has been suggested [38], while other explanations like women of shorter stature having a shorter cervix have been rejected [39].

While being born preterm has received recent recognition as a risk factor for developing hypertension as an adult [40], this is to our knowledge the first time that having mild hypertension (patients with severe hypertension requiring medication were excluded) has been identified as an independent risk factor for SPTB-PPROM with an odds ratio of 9.65 (95% CI 2.5–37.1). Interestingly a family history of recurrent gestational diabetes was associated with SPTB-PPROM, albeit with wide confidence intervals. It is tempting to speculate that the presence of the insulin resistance syndrome would explain these associations [41], [42]. This may also explain the risk associated with hormonal fertility treatment, but again one would typically expect a clear association with the use of clomiphene; an association not demonstrable in this dataset.

It is difficult to explain why waking up during the night would be protective against SPTB-PPROM. Future studies on the full international SCOPE cohort of 5600 women may finally reveal whether this ‘protective’ factor represents a true finding. Similarly, inexplicable at this moment in time, appears to be the risk reduction associated with having a mother who had a miscarriage. Just as surprising was the finding of a doubling of risk associated with the index mother not being the first-born. Thinking of possible suboptimal placentation in the first pregnancy, one would anticipate the opposite.

Variables relating to dental health were only available in just over 30% of recruited women. In these women dental health, as assessed by several specific questions on easily bleeding gums, swollen gums, and sore teeth was no different between women with term birth and women with SPTB-IM or SPTB-PPROM. It should be noted that a recent systematic review [43] on periodontal disease came to an estimated odds ratio of 1.78 (CI 95%: 1.58, 2.01) for SPTB. Our negative findings regarding periodontal health and preterm labour could also be explained by the fact that self-assessed dental health by pregnant women is poorly associated with more objective markers as identified by a professional oral and dental examination [44].

A major strength of this study was its large multicentre prospective design with excellent follow-up. It should be noted that although the current study reports on a large very well defined prospective cohort of more than 3000 healthy nulliparous women, identification of risk factors in the current study risk factor was based on only 156 women with their pregnancies complicated by SPTB. To identify risk factors for very-early preterm birth, much larger prospective cohorts will be required.

### Conclusion

The dissimilarity of clinical risk factors for SPTB-IM compared with SPTB-PPROM indicates different pathophysiological pathways underlie these distinct sub-phenotypes of spontaneous preterm birth. The ability to predict SPTB in healthy nulliparous women using clinical characteristics is modest. Given no reliable biomarkers have emerged as risk predictors of SPTB [45], the development of a clinically useful test will probably require SPTB phenotype-specific combinations of clinical risk factors and the discovery and evaluation of novel biomarkers.

## Supporting Information

Checklist S1 **STROBE Checklist.**
(DOC)

File S1 **Initial variable lists used to train multivariate models.**
(DOC)
