# Supplementary material for: Risk Factors for Preterm Birth in an International Prospective Cohort of Nulliparous Women
Source: PLoS One. 2012 Jul 16;7(7):e39154. doi: 10.1371/journal.pone.0039154 (PMC3398037; doi:10.1371/journal.pone.0039154)
Supplement: File S1 — Initial variable lists used to train multivariate models. (DOC) [file pone.0039154.s002.doc]

**Supporting file 1**

**APPENDIX:** Initial variable list used to train multivariate model

| **SPTB-PPROM** | | | |
| --- | --- | --- | --- |
| 1 | Age | 26 | Sexual intercourse in early pregnancy |
| 2 | Any Accupuncture | 27 | Multiple treatment of Lletz |
| 3 | Taken any marijuana at 1st trim | 28 | Had any of Lletz, Laser or Cryo |
| 4 | Cigarettes 1st Visit | 29 | Colposcopy Treatment Number |
| 5 | Fruit intake 1m prepreg | 30 | Colposcopy last Treatment time >12m |
| 6 | Compare Never to the other groups oth Fish  1 month Pre-preg | 31 | Lletz last Treatment time 7-12m |
| 7 | BMI | 32 | Father deceased |
| 8 | Head Circum | 33 | Any Sister History of Miscarriage |
| 9 | Height | 34 | Combi Sister (More than one sister had) History of PTB_All |
| 10 | Stairs per day | 35 | Family history of recurent GDM |
| 11 | Vigorous Exercise | 36 | Mother Diab Type one |
| 12 | Episodes of waking during the night | 37 | Maternal history of any Miscarriage |
| 13 | Listening Ears | 38 | Number Sisters had Recurrent GDM |
| 14 | Shortest_Trans_Vaginal_Cervical_Length | 39 | Number Sisters had Recurrent GH |
| 15 | Imigration History | 40 | Strong Family history of PET |
| 16 | Ethnicity | 41 | Had Light bleeds and <=6wk |
| 17 | If donor oocyte for this conception | 42 | Had any mod/heavy bleeding>=10 days |
| 18 | History of Infertility | 43 | Had Moderate/Heavy bleeds and >12 wk |
| 19 | In Vitro Fertilization code | 44 | Number of Hospital Admission due to Other Reasons |
| 20 | Months to conceive | 45 | Number of Hospital Admission due to Trauma |
| 21 | Participant's position in family | 46 | Number of Hospital Admission due to Vaginal Bleeding (3 groups) |
| 22 | Fertility treatment Partner or Donor | 47 | Number of Bleeds for >=10days |
| 23 | Hormonal Treatment - Other fertility treatment | 48 | Number of Bleeds, modheavy |
| 24 | Intra-Cytoplasmic Sperm Injection | 49 | Shortest_Trans_Vaginal_Cervical_Length |
| 25 | Mild HT not on antihyp. treat. |  |  |

| **SPTB-IM** | | |
| --- | --- | --- |
| 1 |  | Main Ethncity as Caucasian |
| 2 |  | Current living situation code |
| 3 |  | Participant's gestation at delivery <34wk |
| 4 |  | Any Previous Pregnancy |
| 5 |  | Any Previous Loss with Same partner |
| 6 |  | Any early Pregnancy Loss |
| 7 |  | Months to conceive revised |
| 8 |  | Maternal history of PET |
| 9 |  | Any Sister History of LBW_Baby |
| 10 |  | Strong Family history of LBW_Baby |
| 11 |  | Maternal history of recurrent PTB_All |
| 12 |  | Maternal history of PTB_Spont |
| 13 |  | Family history of PTB_Spont |
| 14 |  | Mother Diab Type two |
| 15 |  | Vaginal Bleeding |
| 16 |  | Number of Bleeds |
| 17 |  | Gestation of 2nd Bleeding |
| 18 |  | Had bleed > 12wk |
| 19 |  | Had any mod/heavy bleeding>=10 days |
| 20 |  | Had spot bleeds and >12 wk |
| 21 |  | Number of Hospital Admission due to Hyperemesis |
| 22 |  | Number of Hospital Admission due to Trauma |
| 23 |  | Cigarettes 1st Visit |
| 24 |  | OtherDrugs 1st trim |
| 25 |  | Marijuana Pre-Preg < 3m |
| 26 |  | Binge_Alc Gest Ceased (Wks of Binge_Alc exposure pre 1st visit) |
| 27 |  | Vigorous Exercise |
| 28 |  | Felt better than ever code |
| 29 |  | 20w Average utri >90th %tile |
| 30 |  | Shortest_Trans_Vaginal_Cervical_Length |
